# Association between individual, household, and area-level socioeconomic status indicators and sensorineural hearing loss in adults in southwest Iran: a population-based study

**DOI:** 10.3389/fpubh.2023.1140500

**Published:** 2023-04-17

**Authors:** Zahra Rahimi, Nader Saki, Bahman Cheraghian, Payam Amini, Masoud Solaymani Dodaran

**Affiliations:** ^1^Department of Epidemiology, School of Public Health, Iran University of Medical Sciences, Tehran, Iran; ^2^Hearing Research Center, Clinical Sciences Research Institute, Ahvaz Jundishapur University of Medical Sciences, Ahvaz, Iran; ^3^Department of Otolaryngology–Head & Neck Surgery, School of Medicine, Ahvaz Jundishapur University of Medical Sciences, Ahvaz, Iran; ^4^Department of Biostatistics and Epidemiology, School of Public Health, Ahvaz Jundishapur University of Medical Sciences, Ahvaz, Iran; ^5^Department of Biostatistics, School of Public Health, Iran University of Medical Sciences, Tehran, Iran

**Keywords:** socioeconomic status, hearing loss, sensorineural, education, Iran

## Abstract

**Introduction:**

Hearing loss is the fourth most common chronic disease, but studies on the relationship between hearing loss and socioeconomic factors are limited. We aimed to examine the association between hearing loss and socioeconomic factors among 35–70 year adults in southwest Iran.

**Materials and methods:**

This population-based cross-sectional study was conducted in the baseline of Hoveyzeh cohort study in adults aged 35–70 in southwest Iran between 2017 and 2021. Information on socioeconomic factors, demographic characteristics, comorbidities, family history of hearing loss, and noise exposure was collected. We assessed the relationship between three levels of socioeconomic factors (individual, household, and area level) with sensorineural hearing loss (SNHL). Multiple logistic regression was used to adjust the potential confounders.

**Results:**

Among a total of 1,365 assessed participants, 485 patients were diagnosed as having hearing loss, and the other 880 individuals were diagnosed without hearing loss, which is considered the case and the control group, respectively. At the individual level of socioeconomic, the odds of having hearing loss in the participants with high school education and diploma, [OR = 0.51 (95%CI:0.28–0.92)], and the individuals with university education [OR = 0.44 (95%CI:0.22–0.87)] were significantly lower than the illiterate participants. At the household socioeconomic level, the odds of having hearing loss were lower for those with poor [OR = 0.63 (95%CI:0.41–0.97)] and moderate [OR = 0.62 (95%CI:0.41–0.94)] wealth status vs. those with the poorest wealth status. In the area level socioeconomic, although the odds of hearing loss in the residents of affluent areas were slightly lower than the residents of deprived areas, there was no significant difference among the groups.

**Conclusion:**

The individuals with hearing loss may have insufficient education and income.

## Introduction

Hearing loss is the most prevalent sensory disorder in the human population (1), which has received much attention in recent years. In 2019, it was reported that 1.57 billion (~20%) people worldwide suffer from hearing loss. Globally, YLDs attributable to hearing loss increased by 73.6% from 25.02 million to 43.45 million between 1990 and 2019 (2). A study in Iran showed that the most common types of disability were “Hearing loss” (68.3%) and “Hearing impairment” (10.4%) (3). Hearing loss can annually impose an economic burden of about $1 trillion (4), and 80% of the global burden of hearing loss is in low- and middle-income countries (LMICs) (5).

Previous research has established health disparities in health outcomes based on socioeconomic status (SES) (6, 7). Despite advances in medicine and technology that have significantly improved human health outcomes, health, and healthcare disparities still exist among communities of different SES (8). However, the exact association mechanism between poor health, socioeconomic burden, and low economic status remains unclear (9).

Otologic problems, including hearing loss, have a direct negative effect on people's daily life, and therefore access to care and treatment services is very important. However, in some countries, including Iran, these services are not evenly distributed and are mostly concentrated in large medical centers. In addition, the provision of screening, treatment, rehabilitation, and rehabilitation services for hearing loss is expensive. Therefore, the frequency of hearing loss is expected to differ in various socioeconomic statuses.

So far, evidence to evaluate the association of hearing loss with socioeconomic factors has been limited. No research has so far been designed to examine the relationship between sensorineural hearing loss (SNHL) and socioeconomic factors in adults in Iran. At the same time, there is an urgent public health need, and understanding this relationship can effectively provide more appropriate recommendations for hearing loss prevention strategies. Therefore, we designed a cross-sectional study to identify the relationship between hearing loss and the socioeconomic factors at the three levels of individual, household, and area socioeconomic status in 35–70 years adults.

## Materials and methods

### Study design and participants

This study was a population-based cross-sectional study conducted in the Hoveyzeh Ear Cohort Study (HECS). HECS is Iran's first cohort study to assess ear health and audiology. It was designed in the context of the Hoveyzeh cohort study (HCS) in southwest Iran, which focused on non-communicable diseases. In the HCS enrollment phase, all eligible people aged 35 to 70 in the region were invited, and 85.16% (1,009 people) accepted the invitation and entered the study (10). We invited the participant from the list of 10,009 individuals who had registered to the Hoveyzeh cohort study according to the order of recruitment date, to perform hearing examinations and fill in the questionnaire. In this study, we recruited 1,872 participants 35–70 years who participated in HECS from Nov 2017 until now. The participants with chronic neurological disorders, inner ear diseases, otosclerosis, taking ototoxic drugs, and mixed hearing loss were excluded from the study. Also, we excluded people with conductive hearing loss (CHL) from the study because conductive hearing loss results from disruption of the transmission of sound through the outer and middle ear and is more common in infants. It can be congenital as a result of an anatomical abnormality, but can also be acquired after an inflammatory disease of the middle ear such as otitis media (11). Finally, 1,365 participants were considered in this analysis. We diagnosed 485 participants with SNHL and 880 individuals without SNHL.

The Ethics Research Committee approved this study of Iran University of Medical Sciences with a code of ethics I.R.IUMS.REC.1399.1441. This study was conducted based on the Helsinki Declaration. Participants signed a written informed consent form for the interview and audiometric evaluation.

### Definition of socioeconomic variables

In this study, we assessed participants‘ socioeconomic status at three levels. Educational levels, resident type, and employment were individual-level socioeconomic indices. Participants' educational levels were categorized as illiterate, primary school, secondary school, high school diploma, and university levels. Residential type is categorized into two groups, rural and urban. Also, the employment variable was defined in the category “Yes” if the participant is employed and “No” if the individual were a housekeeper, retried, or unemployed.

The Wealth score was an indicator of SES at the household level. This index was calculated based on the information on household assets, such as motorcycle, car, TV, cell phone, internet access, vacuum cleaner, freezer, washing machine, computer, and household utilities consisting of house ownership and the number of rooms per capita. A principal component analysis (PCA) was performed to assign a coefficient to each asset. Finally, The wealth scores were recorded into quintiles of poorest, poor, moderate, rich, and richest (12).

The Townsend deprivation index is an area-level SES indicator. This index was calculated using four components in each area: the proportion of households without a car, the proportion of non-homeowner households, the percentage of unemployed residents, and the proportion of overcrowded households. Finally, the computed scores were categorized based on the quintiles as five ordered categories; most affluent, affluent, moderate, deprived, and most deprived (13).

### Other possible confounding factors

We assessed the association between SNHL and demographic variables [sex (male/female)], age groups (35–44, 45–54, 55–64, and ≥65 years), and having comorbidity [hypertension (Yes/No), diabetes (Yes/No)]. Diabetes is defined as a fasting blood glucose level of 126 mg/dl or higher or using glucose-lowering medications or a self-reported diagnosis of diabetes. Hypertension is defined as having systolic blood pressure above 140 mmHg or diastolic blood pressure above 90 mmHg, using blood pressure-lowering medications, or a self-reported diagnosis of hypertension. Also, hearing risk factors were [a family history of hearing loss (Yes/No) and noise exposure (Yes/No)].

### Hearing assessment

A hearing questionnaire was completed for all participants. The information included auditory-vestibular symptoms, noise exposure, and a family history of hearing loss. An experienced audiologist examined the subjects using pure tone audiometry (PTA). Conventional pure tone air conduction (AC) and bone conduction (BC) thresholds are set at octave periods from 250 Hz to 8,000 Hz and 250 Hz to 4,000 Hz, respectively. Normal hearing was determined as the perception of sound stimuli with an intensity of 20 dB HL or less (14) at all frequencies. People with hearing thresholds higher than 20 dB HL in one or more pure tone sounds in both ears were considered having hearing loss. Finally, all diagnoses were confirmed by an otologist.

### Statistical analysis

Descriptive statistics were performed for quantitative variables by mean, standard deviation, and categorical variables by frequency and percentage. The Chi-square test evaluated the association between hearing loss and demographic variables, socioeconomic status, comorbidity, and hearing factors. We examined the independent associations of demographic, clinic, individual, household, and area-level SES with SNHL using multi-level logistic regression, controlling for confounding factors. In the univariate analysis, the criterion for the initial entry of variables into multiple regression models was *P* < 0.25. All reported *p*-values were based on two-tailed tests and considered to have a significance level of 0.05. Stata software version 14.0 was utilized for the statistical analysis.

## Results

In this cross-sectional study, a total of 1,365 participants [Individuals with hearing loss were in the case group (*n* = 485), and those with normal hearing were in the control group (*n* = 880)] were assessed.

Most of the demographic characteristics were significantly different between the two groups. The participants with hearing loss were older (*P* < 0.001) and tended to be male (*P* < 0.001). The family history of hearing loss and noise exposure in the cases group was more reported (*P* < 0.001). Besides, diabetes (*P* < 0.001) and hypertension (*P* < 0.001) were more common in people with hearing loss. Literacy was different between the two groups (*P* = 0.023), so the illiterate participants were more frequent in the case group. A statistically significant difference was seen between the Townsend index and hearing loss (*P* = 0.002), living in the most deprived areas was more prevalent among the cases compared to the controls. On the other hand, there was no significant association between employment, residence type, and wealth score with hearing loss (*P* > 0.05) (Table 1).

**Table 1:** Demographic, socioeconomic, and comorbidity characteristics by hearing status of the participants.

	**Case**	**Control**	*P*-value^*^
	***N*** = **485**, ***n*** **(%)**	***N*** = **880**, ***n*** **(%)**	
**Age category**			
35–44	72 (14.85)	455 (51.70)	<0.001
45–54	162 (33.40)	290 (32.95)	
55–64	185 (38.14)	116 (13.19)	
≥65	66 (13.61)	19 (2.16)	
**Sex**			<0.001
Male	263 (54.23)	261 (29.66)	
Female	222 (45.77)	619 (70.34)	
**Diabetes**			<0.001
Yes	153 (31.55)	149 (16.93)	
No	332 (68.45)	731 (83.07)	
**Hypertension**			<0.001
Yes	182 (37.53)	194 (22.05)	
No	303 (62.47)	686 (77.95)	
**Family history of hearing loss**			<0.001
Yes	51 (10.52)	45 (5.11)	
No	434 (89.48)	835 (94.89)	
**Noise exposure**			<0.001
Yes	141 (29.07)	76 (8.64)	
No	344 (70.93)	804 (91.36)	
**Employment**			0.401
Yes	146 (30.10)	246 (27.95)	
No	339 (69.90)	634 (72.05)	
**Residence type**			0.519
Urban	309 (63.71)	576 (65.45)	
Rural	176 (36.29)	304 (34.55)	
**Education levels**			0.023
Illiteracy	323 (66.60)	508 (57.73)	
Primary school	77 (15.88)	161 (18.30)	
Secondary school	32 (6.60)	73 (8.30)	
High school and Diploma	29 (5.98)	74 (8.41)	
University	24 (4.95)	64 (7.27)	
**Wealth score**			0.230
Poorest	104 (21.44)	145 (16.48)	
Poor	91 (18.76)	177 (20.11)	
Moderate	100 (20.62)	205 (23.30)	
Rich	105 (21.65)	193 (21.93)	
Richest	85 (17.53)	160 (18.18)	
**Townsend index**			0.002
Most affluent	111 (22.89)	256 (29.09)	
Affluent	82 (16.91)	152 (17.27)	
Moderate	93 (19.18)	194 (22.05)	
Deprived	51 (10.52)	92 (10.45)	
Most deprived	148 (30.52)	186 (21.14)	

* Chi-square test.

The Logistic Regression Model was used to estimate crude and adjusted odds ratios. In univariate Logistic Regression analysis, most of the assessed factors, including age, sex, education, Townsend index, Diabetes, Hypertension, Family history of hearing loss, and noise exposure, were significantly associated with hearing loss (*P* < 0.05), while no association found between wealth, employment and type of residence with the disorder (*p* > 0.05). In the next step, all variables with a *p*-value < 0.25 were simultaneously included in the multiple logistic regression model. The findings of the multiple logistic regression analysis showed that the odds of having hearing loss enhanced with increasing the age of participants so that the people in the age group ≥65 years had almost 17 folds more odds of hearing loss in comparison to the participant in the age group 35–44 years [OR =17.93 (95% CI; 9.51–33.82)]. The odds of hearing loss in men were 2.7 times great than in women [OR = 2.70 (95% CI; 1.91–3.83)] (Table 2).

**Table 2:** Crude and adjusted odds ratios of hearing loss using the logistic regression model.

	**Crude odds ratio**	**(CI 95%)**	***P*-value**	**Adjusted odds ratio**	**(CI 95%)**	***P*-value**
**Age groups**						
35–44 y	1			1		
45–54 y	3.53	2.58–4.83	<0.001	3.22	2.26–4.58	<0.001
55–64 y	10.08	7.17–14.16	<0.001	9.03	6.07–13.43	<0.001
≥65 y	17.93	12.44–38.72	<0.001	17.93	9.51–33.82	<0.001
**Sex**						
Male	2.80	2.23–3.53	<0.001	2.70	1.91–3.83	<0.001
Female	1			1		
**Education levels**						
Illiteracy	1			1		
Primary school	0.75	0.55–1.02	0.068	0.77	0.51–1.15	0.198
Secondary school	0.69	0.44–0.1.07	0.096	0.60	0.34–1.08	0.092
High school and Diploma	0.62	0.39–0.97	0.036	0.51	0.28–0.92	0.024
University	0.59	0.36–0.96	0.034	0.44	0.22–0.87	0.019
**Wealth index**						
Poorest	1			1		
Poor	0.72	0.50–1.02	0.07	0.63	0.41–0.97	0.034
Moderate	0.68	0.48–0.96	0.03	0.62	0.41–0.94	0.025
Rich	0.75	0.54–1.07	0.118	0.84	0.54–1.28	0.413
Richest	0.74	0.51–1.1	0.106	0.79	0.49–1.27	0.330
**Townsend index**						
Most deprived	1			1		
Deprived	0.70	0.46–1.04	0.080	0.82	0.50–1.32	0.417
Moderate	0.60	0.43–0.84	0.002	0.87	0.58–1.30	0.490
Affluent	0.68	0.48–0.96	0.027	0.89	0.58–1.37	0.604
Most affluent	0.54	0.40–0.74	<0.001	0.75	0.50–1.14	0.176
**Employment**						
Yes	1.11	0.87–1.42	0.401	-	-	-
No	1			-	-	
**Type of residence**						
Urban	1.07	0.86–1.36	0.519	-	-	-
Rural	1			-	-	
**Diabetes**						
Yes	2.26	1.74–2.93	<0.001	1.56	1.14–2.14	0.006
No	1			1		
**Hypertension**						
Yes	2.12	1.66–2.71	<0.001	1.18	0.87–1.60	0.295
No	1			1		
**Family history of hearing loss**						
Yes	2.18	1.44–3.31	<0.001	2.66	1.62–4.38	<0.001
No	1			1		
**Exposure to noise**						
Yes	4.34	3.19–5.89	<0.001	3.09	2.11–4.52	<0.001
No	1			1		

**Figure 1:**
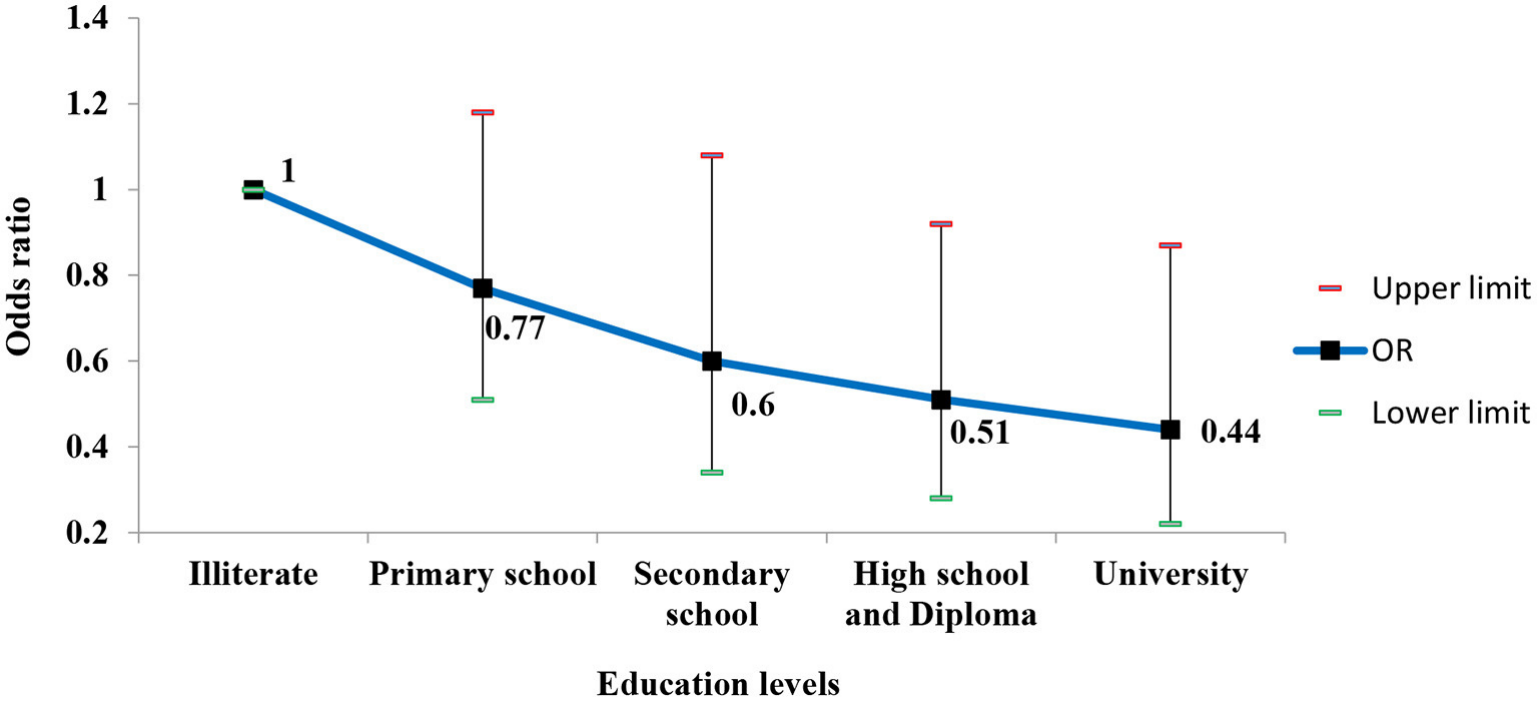
Adjusted odds ratios (95% CI) of hearing loss according to levels of education.

Among the three assessed socioeconomic factors, education level had an inverse and significant association with hearing loss (*P* = 0.004), so the odds of hearing loss continuously decreased with education levels (Figure 1).

**Figure 2:**
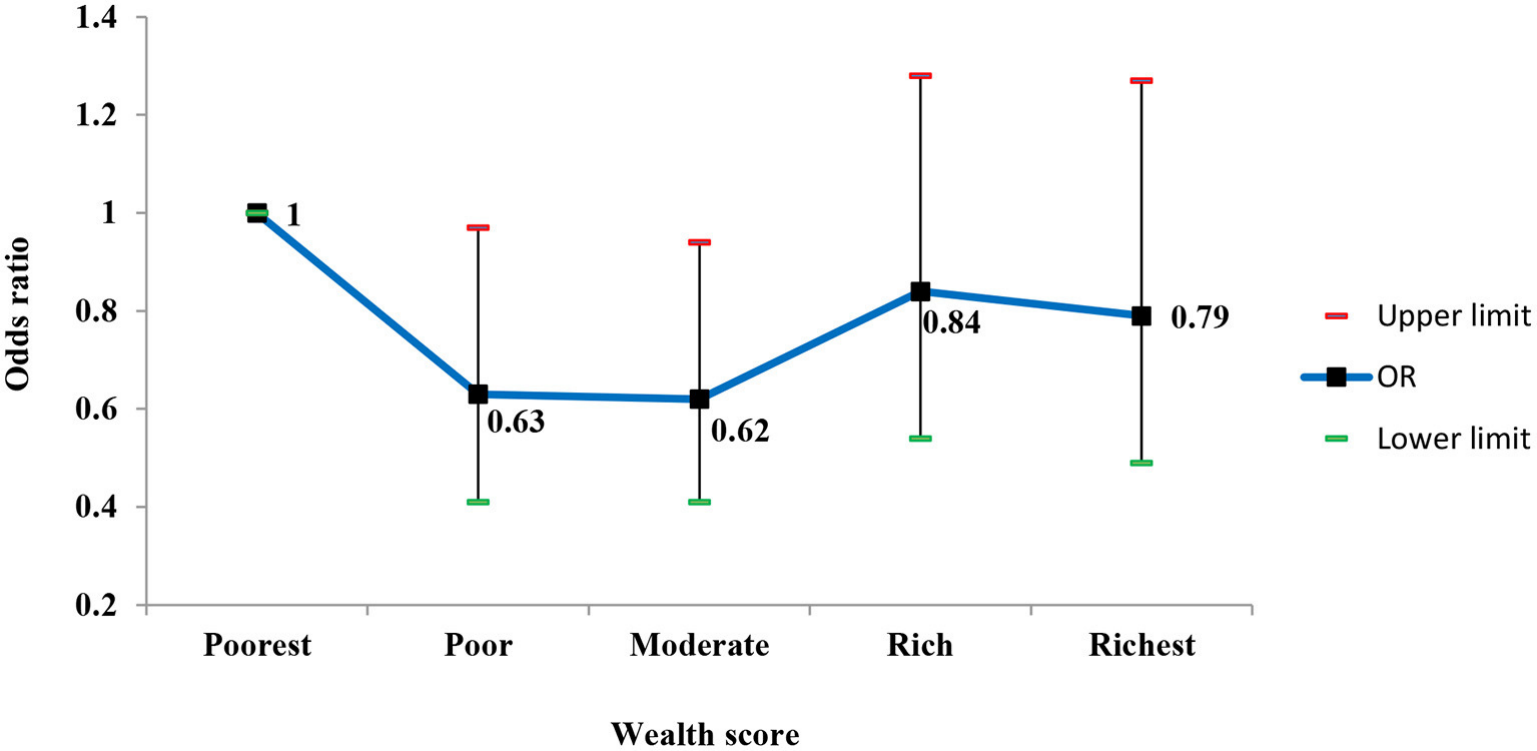
Adjusted odds ratios (95% CI) of hearing loss according to wealth status.

This result indicated that the odds of having hearing loss in the participants with university education was about two times less than illiterate participants (reference group) [OR = 0.44 (95% CI; 0.22–0.87)]. Overall, the odds of having hearing loss decreased with increasing the wealth status of the participants; so it was statistically lower in participants in the poor and moderate quintiles in comparison to the poorest (Figure 2).

**Figure 3:**
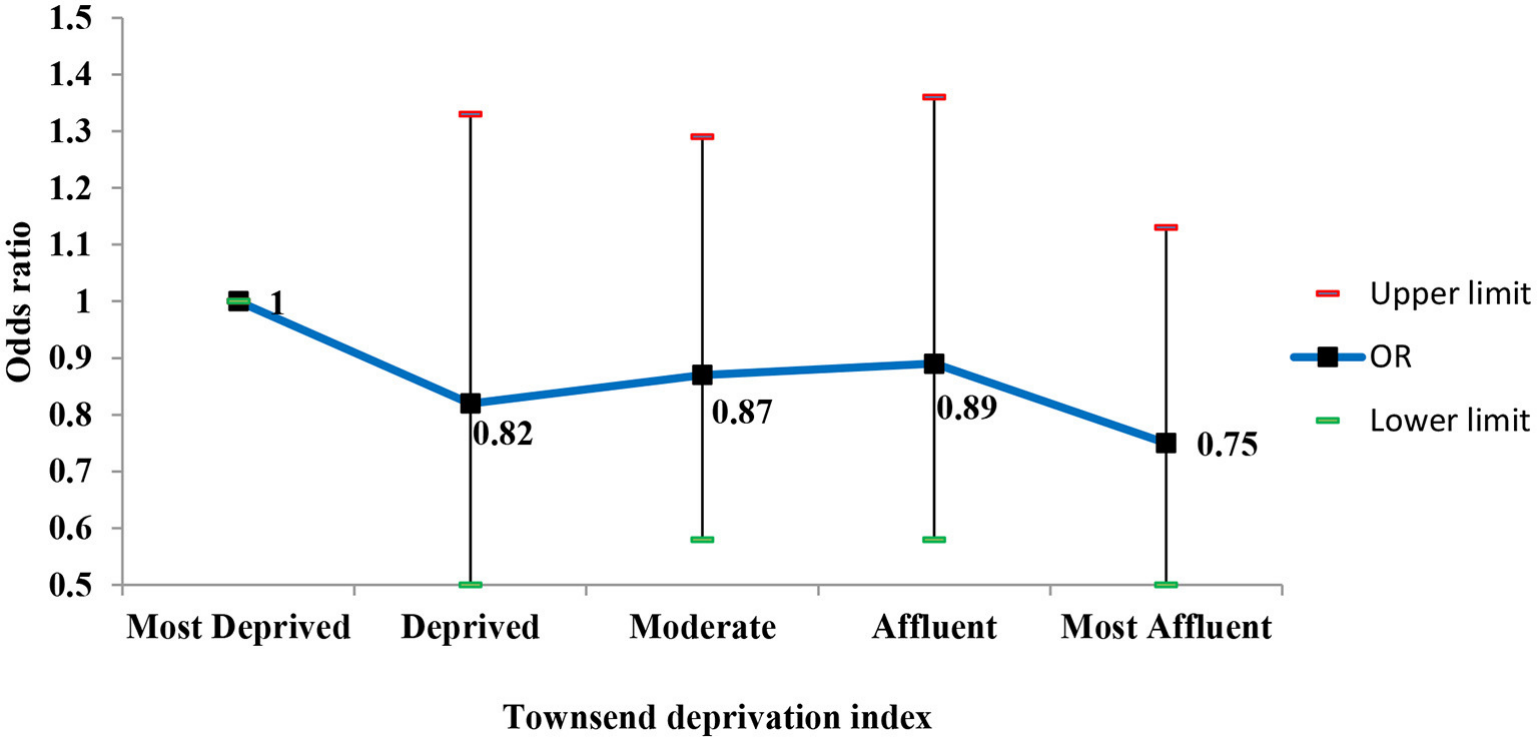
Adjusted odds ratios (95% CI) of hearing loss according to the Townsend deprivation index.

Although the odds of having hearing loss in the residents of affluent areas were lower than in those living in the deprived areas, after adjusting for confounding factors, this association was not statistically significant (Figure 3).

Moreover, the results showed a significant association between diabetes and hearing loss (*P* = 0.006). The diabetic participants had 56 percent more hearing loss than the non-diabetic people [OR = 1.56 (95% CI; 1.14–2.14)]. The participants with a family history of hearing loss were 2.5 times more likely to have hearing loss than those without a family history [OR =2.66 (95% CI; 1.62–4.38)] and this association was statistically significant (*P* < 0.001). Also, a statistically significant association was found between exposure to noise and hearing loss (*P* < 0.001), so the individual with noise exposure had threefold more odds of hearing loss than the unexposed group [OR =3.09 (95% CI; 2.12–4.52)]. However, there was no significant association between hypertension and hearing loss (*P* = 0.295).

## Discussion

### Summary of main findings

We found that education level as an individual socioeconomic level was statistically associated with SNHL, which is an important determinant of hearing loss. At the household level, hearing loss was more prevalent in the poorest households. Besides, at the area level, hearing loss was more prevalent in the most deprived areas, although, there was no statistically significant relationship between SNHL and Townsend Deprived Index. Our findings demonstrated strong associations between hearing loss and sex, age, diabetes, noise exposure, and family history of hearing loss.

### Comparison of our findings with previous studies

This study showed a direct relationship between age and hearing loss. Most studies mentioned that age was significantly associated with hearing loss, indicating that older adults were at higher risk than younger adults (15–20). Some believe that progressive hearing loss occurs as a normal outcome of aging (21) due to a slow progressive decline in the ability to perceive high-frequency tones, caused by the degeneration of the hair cells in the ear (22). Also, aging may lead to the accumulation of oxidative stress damage, mitochondrial DNA damage, and the induction of cochlear cell apoptosis and, eventually, hearing loss (23, 24). Another explanation may be due to the exposure accumulation of the risk factor of deleterious auditory the during the aging process (25).

In our study, hearing loss was more common in men, even after adjustment for the effects of other variables. Most studies reported men had higher odds of hearing loss (18–21, 26, 27). It might reflect gender differences in exposure to other hearing loss risk factors such as lifestyle and CVD.

Our result showed that education level was inversely associated with hearing loss. The results of some studies were also in line with our findings (26, 28–30). A low level of education may be related to an unhealthy lifestyle (31), such as smoking and noise exposure, which may lead to a higher risk of hearing loss. Individuals with higher education levels may have better nutrition, access to health care, fewer ear infections, or more usage of hearing protection devices. However, the relationship between hearing loss and fewer years of formal schooling may be subject to reverse causality bias. Hearing loss can provide some limitations for people that prevent continuing education and lead to low educational levels (21).

We observed an inverse gradient between hearing loss and the wealth index. Participants in lower wealth quintiles experienced significantly high occurrence of hearing loss compared to those in the highest wealth quintile. Perhaps the reason is that in the poorest households, most of their income is spent on daily expenses, including the purchase of food, and the share of income that can be spent on health care is limited.

Studies on the relationship between area-level socioeconomic indicators and hearing loss are scarce. Our results demonstrated an inverse but non-significant relationship between the Townsend deprivation index as an area-level socioeconomic indicator and hearing loss so that people who lived in the most deprived areas experienced hearing loss more than those living in the most affluent areas. This finding was consistent with the previous results on the relationship between regional SES and chronic disease (6). The association of regional SES and multiple health outcomes is mediated or influenced by several factors: disparities in access to social and health resources, to the detriment of environmental exposure (e.g., noise and overcrowding), difficult living conditions, cost of available resources, etc. On the contrary, a Chinese study showed that people living in affluent areas had more hearing impairment than those in deprived areas and reported loud noise above 80 dB caused by heavy traffic in affluent areas of China (25).

Most previous studies have shown that hearing impairment is associated with lower income (9, 32). Low income might be related to various causes of hearing impairment, including ear infections, lifestyle factors such as smoking (33), lax enforcement of noise exposure regulations, and a lack of access to preventive hearing care and health services (34). Some studies reported that lack of health insurance was the main reason for the lack of access to health care services (9). Because hearing screening and audiology evaluation services are expensive, access to these services is difficult for low-income people. In addition, we found that unemployment was higher in people with hearing loss, which agrees with the previous reports (29, 30). Unemployment can affect poor hearing knowledge and less access to health care for hearing impairments (29, 35).

According to several studies, hearing loss in people with type 2 diabetes was more prevalent (19, 36–38), which was in line with the results of our research. Some mechanisms of hearing loss in diabetic patients include microangiopathy of the inner ear, neuropathy of the cochlear nerve, outer hair dysfunction, and disruption of the endolymphatic potential (39). The mechanisms of the effect of hypertension on hearing loss are not yet clear (40). It has been hypothesized that common cardiovascular risk factors, such as hypertension through damage to the cochlear microvasculature, may play an essential role in the etiology of hearing loss (31). However, in our study, after adjustment for demographic and socioeconomic factors, comorbidities, history of noise exposure, and history of hearing loss in the family, there was no statistically significant association between hypertension and hearing loss; similar results were reported from the other studies (31, 41).

We showed a statistically significant relationship between hearing loss and a family history of hearing loss. Recently, evidence of gene-environment interactions in adult hearing loss has accumulated (42). Several studies have shown a strong relationship between hereditary hearing loss and being siblings, cousins, or common family ancestry (43–45). The pairing of hearing loss genes is more common in relatives. Besides, a positive family history of hearing loss can result from common cultural and lifestyle characteristics for family members. We suggest a direct relationship between noise exposure and hearing loss. Previous evidence confirmed the role of noise exposure in the onset or progression of hearing impairment (27, 46) through neuropathy of the end of the cochlea auditory nerve (47).

### Strengths and limitations

Our study has several strengths, including conducting this study in the context of a population-based cohort study using a representative sample of the population that can reduce selection bias; examining the relationship of SES at three individual, household, and regional levels separately; applying valid instruments and measure audiometric tests according to the definition of the world health organization.

There were some limitations in our study. First, the relationship between hearing loss and noise exposure may be susceptible to recall bias. Hence, the patients with hearing loss are more likely to recall and report a history of noise exposure than the control group. This can overestimate the reported odds ratios. Second, considering the study design, exposure-outcome temporality cannot be demonstrated. Hence, the relationship between hearing loss and literacy may be subject to reverse causality bias.

## Conclusion

Our findings revealed that SNHL was inversely related to SES. Among the three assessed socioeconomic indicators, education level and wealth index had the association with hearing loss. This shows that individual and household socioeconomic indicators are more associated with hearing loss. Understanding the socioeconomic impact of hearing loss can help health policymakers in planning health improvement programs, including screening and educational interventions. Besides, early diagnosis of hearing loss in high-risk people, treatment and rehabilitation measures were performed earlier and more effectively. The relationship between socioeconomic status and hearing loss is complex, likely involving multiple simultaneous pathways. Therefore, future longitudinal studies will be necessary to better understand the mechanisms behind these associations.

## Data availability statement

The original contributions presented in the study are included in the article/supplementary material, further inquiries can be directed to the corresponding author.

## Ethics statement

The studies involving human participants were reviewed and approved by the Vice-Chancellor provided financial support for Research at Iran University of Medical Sciences, Tehran, Iran. The patients/participants provided their written informed consent to participate in this study.

## Author contributions

MS, NS, BC, PA, and ZR contributed to the design and preparation of the manuscript and its interpretation. ZR was involved in the data collection and perforation. PA, BC, and ZR performed the statistical analysis. All authors have reviewed and approved the manuscript.
